# Surgical management of right hepatectomy after coronary artery bypass grafting using the right gastroepiploic artery: a case report and literature review

**DOI:** 10.1186/s12957-024-03401-w

**Published:** 2024-05-03

**Authors:** Nao Kawaguchi, Shun Kizawa, Masahiro Daimon, Hiroki Minami, Yasuhiko Ueda, Atsushi Tomioka, Koji Komeda, Mitsuhiro Asakuma, Hideki Tomiyama, Sang-Woong Lee

**Affiliations:** 1 Department of General and Gastroenterological Surgery, Osaka Medical and Pharmaceutical University , 2-7 Daigaku-machi, Takatsuki, 569-8686 Osaka Japan; 2 Department of Cardiology, Takatsuki Red Cross Hospital, 1-1-1 Abuno, Takatsuki, 569- 1096 Osaka Japan; 3 Department of Thoracic and Cardiovascular Surgery, Osaka Medical and Pharmaceutical University, 2-7 Daigaku-machi, Takatsuki, 569-8686 Osaka Japan

**Keywords:** Coronary artery bypass grafting, Right gastroduodenal artery graft, Right hepatectomy, Hepatic falciform ligament

## Abstract

**Background:**

Coronary artery bypass grafting (CABG) using the right gastroepiploic artery (RGEA) is a well-established, safe procedure. However, problems with RGEA grafts in subsequent abdominal surgeries can lead to fatal complications. This report presents the first case of right hepatectomy for hepatocellular carcinoma after CABG using the RGEA.

**Case presentation:**

We describe a case in which a right hepatectomy for an 81-year-old male patient with hepatocellular carcinoma was safely performed after CABG using a RGEA graft. Preoperatively, three-dimensional computed tomography (3D- CT) images were constructed to confirm the run of the RGEA graft. The operation was conducted with the standby of a cardiovascular surgeon if there was a problem with the RGEA graft. The RGEA graft had formed adhesions with the hepatic falciform ligament, necessitating meticulous dissection. After the right hepatectomy, the left hepatic lobe descended into the vacated space, exerting traction on the RGEA. However, this traction was mitigated by suturing the hepatic falciform ligament to the abdominal wall, ensuring stability of the RGEA. There were no intraoperative or postoperative complications.

**Conclusion:**

It is crucial to confirm the functionality and anatomy of the RGEA graft preoperatively, handle it gently intraoperatively, and collaborate with cardiovascular surgeons.

## Background

The right gastroepiploic artery (RGEA) is used in coronary artery bypass grafting (CABG) [1, 2]. However, patients who undergo CABG with an RGEA are at risk of pedicle injury during subsequent abdominal surgeries. Various previous reports on abdominal surgery, such as gastrectomy, cholecystectomy, and pancreatoduodenectomy in patients with RGEA grafts for coronary bypass, have been reported [3–6]. This report presents the first case of right hepatectomy after CABG using the RGEA. Injury or spasm of the RGEA graft can lead to life-threatening myocardial ischemia. Consequently, a comprehensive strategy is required during the perioperative period to avoid risks while preserving the operation’s curative potential.

## Case presentation

**Fig. 1:**
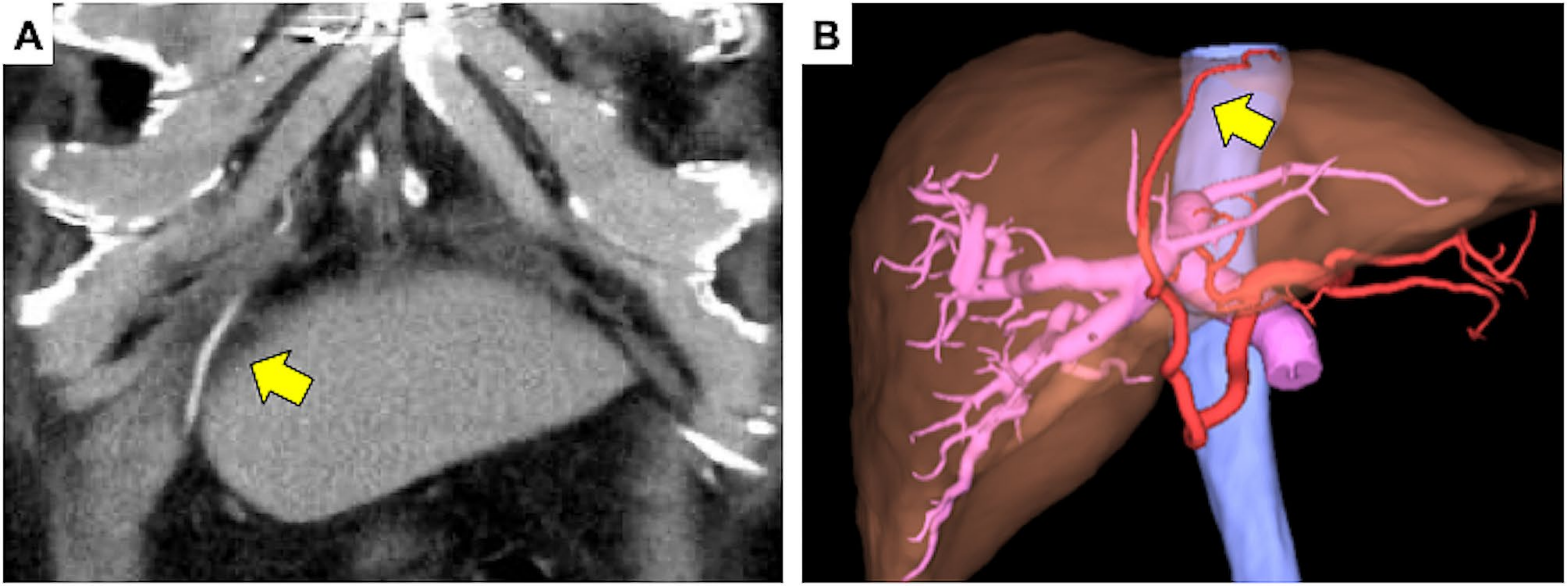
Enhanced abdominal computed tomography (CT) and SYNAPSE VINCENT results. Enhanced CT (**A**) and SYNAPSE VINCENT (**B**) shows the right gastroepiploic artery graft along the hepatic falciform ligament (arrow). CT: computed tomography

**Fig. 2:**
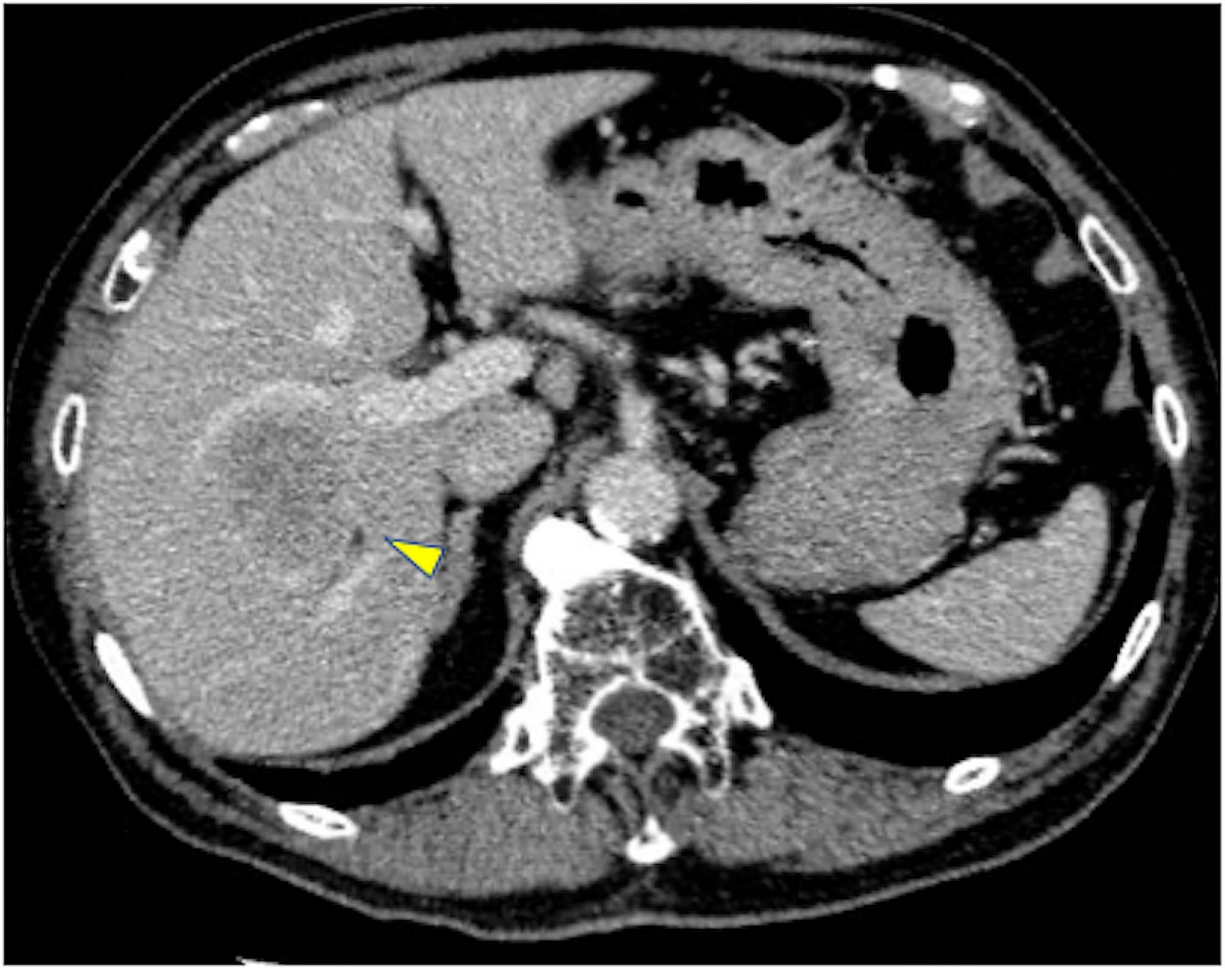
Enhanced abdominal computed tomography (CT) results. Enhanced CT shows a large solitary tumor measuring 6.0 cm located near the bifurcation of the anterior and posterior hepatic branches (arrowhead). CT: computed tomography

We present the case of an 81-year-old male patient with a history of a CABG using the RGEA. He had a past medical history of diabetes mellitus and the lower extremity arteriosclerosis obliterans. Preoperative enhanced computed tomography (CT) imaging revealed an enlarged hepatic tumor and a patent RGEA graft was observed on the left lobe of the liver (Fig. 1). Subsequent liver biopsy confirmed the diagnosis of hepatocellular carcinoma as a consequence of nonalcoholic steatohepatitis. The tumor was located near the bifurcation of the anterior and posterior hepatic branches (Fig. 2), warranting a right hepatectomy. As the percentage of the remaining liver was only 41%, preoperative transhepatic portal vein embolization (PTPE) was initially performed to promote enlargement of the left lobe. This procedure increased the proportion of the remaining liver to 45%, after which an open right hepatectomy was planned. The Child-Pugh classification, which reflects preoperative liver function, was A (5 points), and the indocyanine green(ICG) retention test after 15 minuets was 1.0%.

**Fig. 3:**
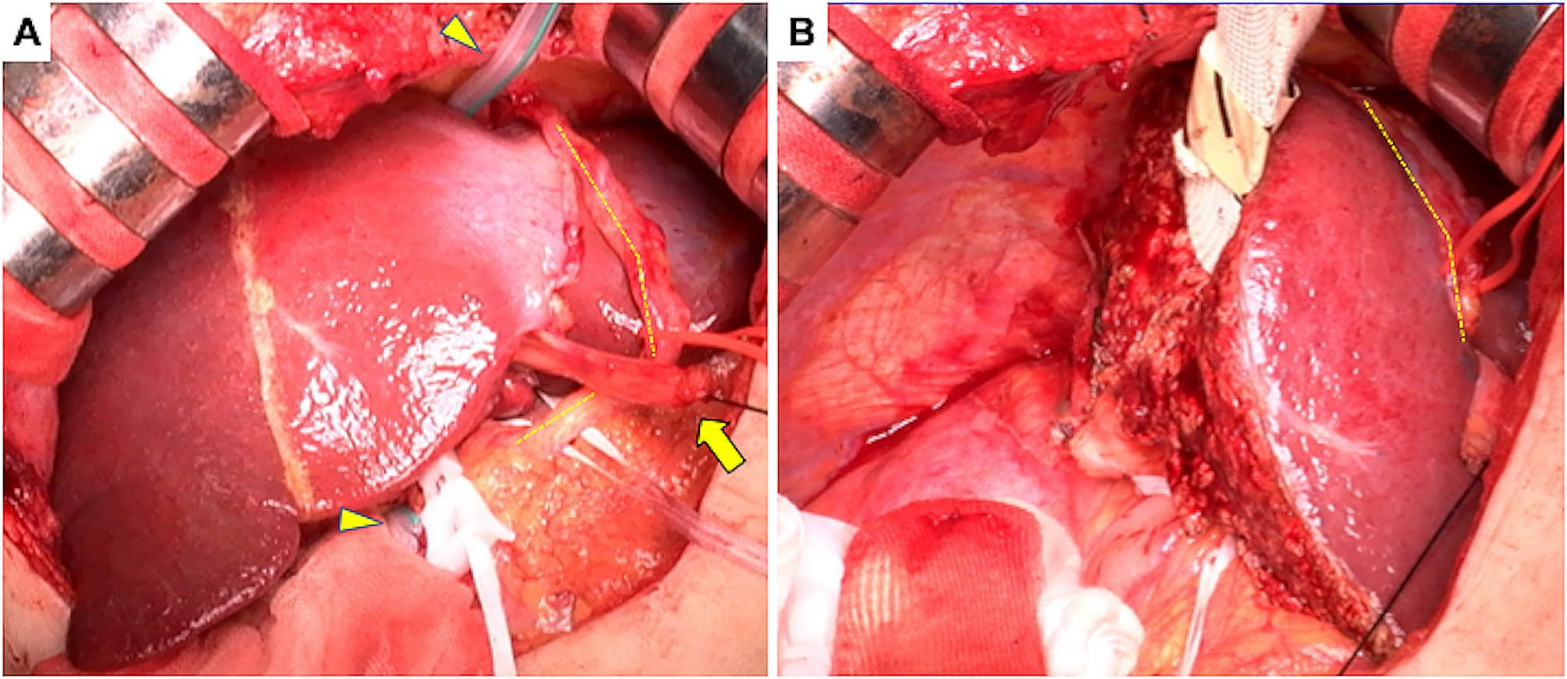
Intraoperative findings Yellow dotted line indicates the RGEA. (**A**) The RGEA graft tightly adheres to the falciform ligament. The technique of liver hanging maneuver is used with Penrose drain (arrowhead). (**B**) Right hepatectomy is performed. RGEA: right gastroepiploic artery

A median skin incision with right transverse extension was performed. A wound protector was not used to prevent potential damage to the RGEA. The RGEA graft coursed past the ventral side of the stomach to the left of the hepatic falciform ligament and proceeded along the ventral side of the liver into the heart. The RGEA graft tightly adhered to the falciform ligament, meticulously separated from the liver’s surface, and was secured (Fig. 3A). The RGEA graft was taped, taking the utmost care not only to prevent any intraoperative damage but also to ensure that no unnecessary tension was applied.

**Fig. 4:**
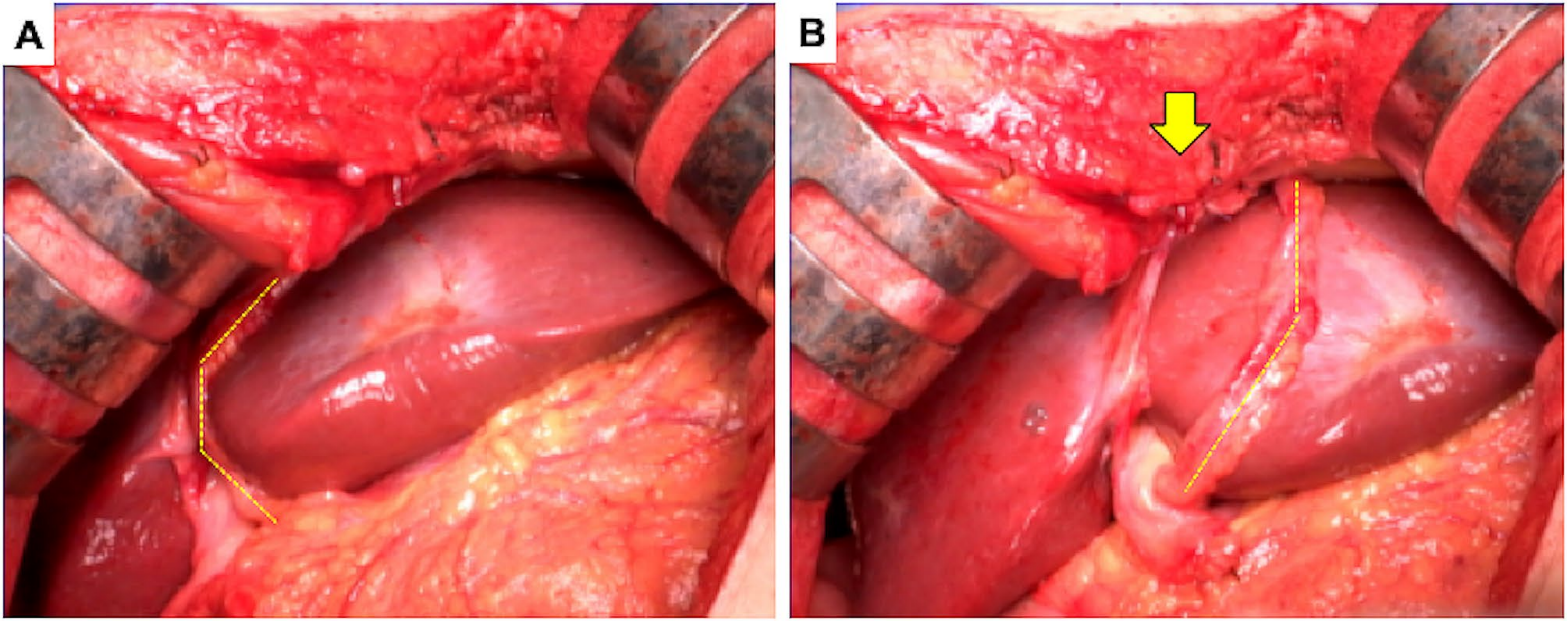
Intraoperative findings. Yellow dotted line shows the RGEA. (**A**) The RGEA is towed because the left lobe of the liver had moved to the right temporal side. (**B**) The falciform ligament is anchored to the abdominal wall to avoid tension exerted by the RGEA (arrow). RGEA: right gastroepiploic artery

**Fig. 5:**
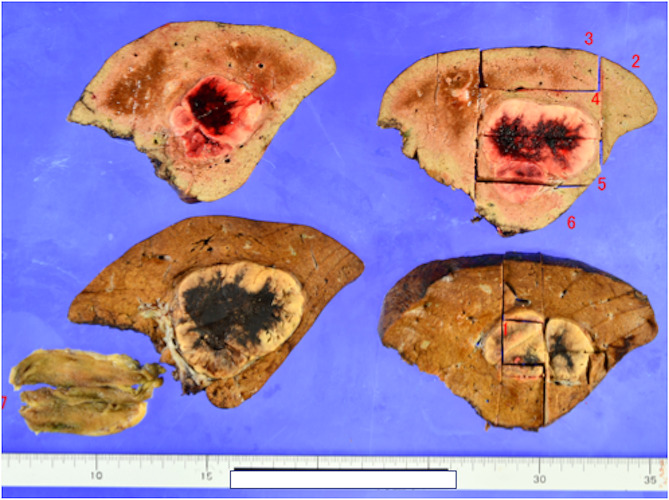
Histopathological specimen result. The liver tumor is a moderately differentiated hepatocellular carcinoma; im(-), e.g., fc(-), fc-inf(-), sf(-), s0, vp0, vv0, va0, b0, p0, sm(-), f1

Following the detachment of the RGEA graft from the liver, a right hepatectomy was performed (Fig. 3B). The left lobe was retracted to the new space of the right temporal side, which might have exerted tension on the RGEA graft; therefore, the falciform ligament was anchored to the abdominal wall (Fig. 4). Consequently, traction on the RGEA graft was avoided. No cardiac events occurred during the right hepatectomy, with a total intraoperative blood loss of 175 ml over a total operative time of 268 min. The resected specimen weighed 600 g, and the tumor measured 6.0 × 5.0 cm. Histopathological examination revealed a moderately differentiated hepatocellular carcinoma (Fig. 5). Continuous preoperative heparin was administered to ensure optimal circulation through the RGEA graft. Heparin was discontinued 6 h before surgery and resumed the day following the procedure. The patient’s postoperative course was uneventful, except that diuretics were started due to ascites. The patient was discharged on the 19th postoperative day. One and a half years after the surgery, the patient showed no recurrence of hepatocellular carcinoma or cardiovascular events.

## Discussion

RGEA grafts are excellent conduits for coronary revascularization, similar to the internal thoracic artery. Long-term patency of RGEA grafts as conduits for CABG has also been reported [1, 2, 7]. The RGEA is currently used as a bypass graft for posterior coronary circulation. However, CABG using RGEA is not often performed. In the presented case, the right coronary artery exhibited significant stenosis (99% occlusion). Additionally, the ascending aorta’s condition was deemed suboptimal. To minimize manipulation of the ascending aorta and considering the patient’s atherosclerosis obliterans in the lower limbs and diabetes mellitus, conserving the saphenous vein was a prudent decision. The patient’s lower limb wounds might be challenging to heal, necessitating the retention of the saphenous vein in anticipation of potential future arterial bypass requirements due to the progression of arteriosclerosis obliterans. For these reasons, the RGEA graft was used. Graft injuries during abdominal surgery after CABG using the RGEA may cause coronary failure and fatal arrhythmia. To avoid graft injury, it is important to understand how to harvest and place the graft in CABGs. There are two possible routes for RGEA grafting: ante-gastric and retro-gastric. Most cardiac surgeons employ the ante-gastric route because it allows easier identification of bleeding from the RGEA pedicle. In this case, the graft traversed the ventral side of the stomach, proceeded to the left side of the hepatic falciform ligament via the liver’s ventral surface, and reached the heart through an opening in the diaphragm (Fig. 1).

During laparotomy, the RGEA frequently adheres to the abdominal wall, hepatic falciform ligament, and liver surface. In the present case, it adhered to the liver surface adjacent to the hepatic falciform ligament. 　Because of the adhesion of the graft to the hepatic dissection line, we were forced to remove the graft from the hepatic surface, but we were able to do so without damaging the graft. Fortunately, no electrocardiographic changes were observed during the dissection or taping. However, spasms and injuries induced by graft handling during surgery can cause critical coronary failure [3]. To avoid inadvertent graft injury, in addition to perioperative management with anticoagulation therapy, an understanding of the graft route and careful and conservative surgical manipulation are necessary.

Various reports on abdominal surgery after CABG with the RGEA have been published. The following is a summary of the points to be considered for each surgery. Sakamoto et al. reported that in laparoscopic cholecystectomy, during dissection of Calot’s triangle, it is necessary to ensure that the pedicle is not stretched, and it is important to use a lower pneumoperitoneal pressure (such as 5 mmHg). In laparoscopic colectomy, it has also been reported that care should be taken to avoid the possibility of unexpected excessive stress on the RGEA graft due to positional changes [8, 9]. Although gastrectomy is most frequently reported, the RGEA graft may be resected, and a replacement graft may be used from the viewpoint of the lymph node dissection. However, in cases such as early stage cancer, there is little metastasis to the lymph nodes, and RGEA resection should be performed with caution [3]. There are reports that pancreaticoduodenectomy allows grafts to be left in place [5], and there are also reports of preoperative reconstruction of a replacement graft [6, 10]. Hepatic resection is more variable depending on the site of resection; however, more caution is required for left lobectomy because the graft often runs over the anterior aspect of the left lobe [11]. The two-stage explantation method has been reported to be effective in preserving RGEA grafts during transplantation [12]. There have also been reports of a backup system for cardiovascular surgery and the placement of a sheath in the femoral artery for quick response [3]. Moreover, a recent report showed that real-time vessel navigation using indocyanine green fluorescence (ICG) and visualization of the RGEA graft is useful for easy identification [13].

In this case, the left hepatic coronary mesentery was not dissected, but the RGEA was in traction, as the left lobe of the liver fell into the space after right lobectomy. Therefore, the falciform ligament was anchored to the abdominal wall to prevent tension. Laparoscopic surgery has increased in recent years and can be performed in the left semi-supine position. In such situations, it is essential to verify the condition of the RGEA graft with the patient in a supine position. Furthermore, securing it to the abdominal wall is crucial for preventing complications associated with RGEA grafts. In the future, with the increasing number of patients with metabolic syndrome, particularly hyperlipidemia, the likelihood of performing abdominal surgery after CABG using RGEA grafts, as in our case, is expected to increase. Gaining a thorough understanding of the preoperative anatomy and being cognizant of the pitfalls and innovations in each surgical procedure are paramount.

## Conclusion

To prevent damage to the RGEA graft, a comprehensive understanding of its’ anatomy before surgery is crucial. In right lobectomy, it is essential to secure the left lobe of the liver to the abdominal wall using the falciform ligament to minimize the tension on the RGEA. Collaboration with cardiovascular surgeons is of the utmost importance.

## Data Availability

No datasets were generated or analysed during the current study.

## Abbreviations

CABG: Coronary artery bypass grafting
CT: Computed tomography
PTPE: Preoperative transhepatic portal vein embolization
RGEA: Right gastroepiploic artery
